# Topics of Public Interest

**Published:** 1904-05

**Authors:** 


### TOPICS OF PUBLIC INTEREST.

THE passing of the coroner in Greater New York will soon be witnessed. Senator Elsberg introduced a bill early in the session of the last legislature to substitute medical examiners for coroners on the expiration of the terms of the latter officers and under the whip and spur of an emergency message from Governor Odell, which seemed to be necessary to arouse the members to the importance of the measure, the bill, having previously received the sanction of the senate, was passed in the assembly by a vote of 80 to 30.

The new system established is substantially that which has been in vogue in Buffalo and the county of Erie for some years, and we cannot doubt that New York will experience, in the substitution of qualified medical examiners for political place holders, similar benefits to those already realised here.

---

Dr. C. G. LEO WOLF, health officer of Niagara Falls, has submitted his report for 1903, from which some interesting figures are obtained. For example, we observe that the population of the city of the cataract has increased more than 10,000 in the last five years. The number of births decreased during 1903 by 29, whereas the number of marriages was 65 more than during the previous year, and the decrease in the number of deaths was 11. Typhoid fever is on the increase, 14 more deaths being recorded for 1903 than for 1902. The entire report is most creditable to the health officer, indicating efficiency in administration and economy of expenditures.

